# *PER3* Polymorphism Predicts Cumulative Sleep Homeostatic but Not Neurobehavioral Changes to Chronic Partial Sleep Deprivation

**DOI:** 10.1371/journal.pone.0005874

**Published:** 2009-06-11

**Authors:** Namni Goel, Siobhan Banks, Emmanuel Mignot, David F. Dinges

**Affiliations:** 1 Division of Sleep and Chronobiology, Department of Psychiatry, University of Pennsylvania School of Medicine, Philadelphia, Pennsylvania, United States of America; 2 Center for Narcolepsy, Department of Psychiatry and Behavioral Sciences, and Howard Hughes Medical Institute, Stanford University, Palo Alto, California, United States of America; Pennsylvania State University, United States of America

## Abstract

**Background:**

The variable number tandem repeat (VNTR) polymorphism 5-repeat allele of the circadian gene *PERIOD3* (*PER3^5/5^*) has been associated with cognitive decline at a specific circadian phase in response to a night of total sleep deprivation (TSD), relative to the 4-repeat allele (*PER3^4/4^*). *PER3^5/5^* has also been related to higher sleep homeostasis, which is thought to underlie this cognitive vulnerability. To date, no study has used a candidate gene approach to investigate the response to chronic partial sleep deprivation (PSD), a condition distinct from TSD and one commonly experienced by millions of people on a daily and persistent basis. We evaluated whether the *PER3* VNTR polymorphism contributed to cumulative neurobehavioral deficits and sleep homeostatic responses during PSD.

**Methodology/Principal Findings:**

*PER3^5/5^* (n = 14), *PER3^4/5^* (n = 63) and *PER3^4/4^* (n = 52) healthy adults (aged 22–45 y) demonstrated large, but equivalent cumulative decreases in cognitive performance and physiological alertness, and cumulative increases in sleepiness across 5 nights of sleep restricted to 4 h per night. Such effects were accompanied by increasing daily inter-subject variability in all groups. The *PER3* genotypes did not differ significantly at baseline in habitual sleep, physiological sleep structure, circadian phase, physiological sleepiness, cognitive performance, or subjective sleepiness, although during PSD, *PER3^5/5^* subjects had slightly but reliably elevated sleep homeostatic pressure as measured physiologically by EEG slow-wave energy in non-rapid eye movement sleep compared with *PER3^4/4^* subjects. *PER3* genotypic and allelic frequencies did not differ significantly between Caucasians and African Americans.

**Conclusions/Significance:**

The *PER3* VNTR polymorphism was not associated with individual differences in neurobehavioral responses to PSD, although it was related to one marker of sleep homoeostatic response during PSD. The comparability of *PER3* genotypes at baseline and their equivalent inter-individual vulnerability to sleep restriction indicate that *PER3* does not contribute to the neurobehavioral effects of chronic sleep loss.

## Introduction

Subjects undergoing total sleep deprivation (TSD) display differential vulnerability to sleep loss, demonstrating robust inter-individual differences in response to the same laboratory conditions, as measured by various neurobehavioral tasks and physiological and subjective sleep measures sensitive to sleep loss [1]–[6]. Some of these measures, including self-rated sleepiness, habitual sleep duration and habitual bedtime show moderate heritability [7]. Such differential vulnerability extends to chronic partial sleep deprivation (PSD) protocols, in which sleep is restricted to 3-7 h time in bed per night [8], [9]. The stable, trait-like inter-individual differences observed in response to TSD [1]–[4]—with intraclass correlations ranging from 58–92% for neurobehavioral measures [1], [4]—point to an underlying genetic component.

Despite this link, however, little is known about the genetic basis of differential vulnerability to sleep loss on functioning in healthy subjects undergoing deprivation [10], [11]. Recently, two related publications reported on the role of the variable number tandem repeat (VNTR) polymorphism of the circadian gene *PERIOD3* (*PER3)* in response to TSD by using a small group of subjects (n = 24 of various ethnicities) recruited specifically for the homozygotic versions of this polymorphism. *PER3* is characterized by a 54-nucleotide coding region motif repeating in 4 or 5 units and shows similar allelic frequencies in African Americans and Caucasians/European Americans [12], [13]. Compared with the 4-repeat allele (*PER3^4/4^*; n = 14), the longer, 5-repeat allele (*PER3^5/5^*; n = 10) was associated with worse cognitive performance and with higher sleep propensity including slow-wave activity in the sleep EEG—a putative marker of sleep homeostasis—before and after TSD [14]. A subsequent report on the same subjects clarified that the *PER3^5/5^* performance deficits were found only on specific executive function tests, and only at 2–4 h following the melatonin circadian rhythm peak, from approximately 6–8 am [15]. Such performance differences were hypothesized to be mediated by sleep homeostasis [14], [15].

Since the *PER3* VNTR polymorphism predicted individual differences in sleep-loss-induced decrements in performance and in sleep homeostasis following a night of TSD [14], [15], we sought to determine whether this polymorphism was also associated with cumulative performance deficits and sleep homeostatic responses during chronic PSD—a condition experienced by millions of people on a consecutive and daily basis [16]–[18], associated with serious health consequences [16], [19], and distinguished from TSD [8], [20]. Because of this distinction, different genetic polymorphisms may influence responses to TSD and chronic PSD. To date, however, no published study has examined the *PER3* VNTR or any other genetic polymorphism and its relationship to cumulative neurobehavioral or homeostatic functioning in chronic PSD.

In the current study, the *PER3* VNTR polymorphism was not associated with individual differences in neurobehavioral responses to PSD, although it was related to one marker of sleep homoeostatic response. Equivalent baseline physiological, cognitive and subjective measures and comparable inter-individual vulnerability to PSD indicate genes other than *PER3* contribute to the cumulative behavioral effects of chronic partial sleep loss.

## Results

Fifty-two *PER3^4/4^* (M = 31.2 y±7.0 y; 24 women, 20 Caucasians, 31 African Americans), 63 *PER3^4/5^* (M = 28.9 y±6.3 y; 33 women, 24 Caucasians, 37 African Americans) and 14 *PER3^5/5^* (M = 29.7 y±8.2 y; 9 women, 5 Caucasians, 8 African Americans) healthy adults were characterized for their cognitive performance, self-rated sleepiness, executive functioning and physiological sleep responses to a PSD protocol of 4 h time in bed for five consecutive nights.

### Demographic and Pre-study Variables

As shown in Table 1, the three *PER3* genotypes did not differ significantly in demographic variables including age, body mass index, or sex, or in ethnicity, in terms of the genotypic and allelic frequency between Caucasians and African Americans. Moreover, the groups did not differ in IQ as measured by the North American Adult Reading Test [21], or in psychosocial/personality traits, as measured by the Beck Depression Inventory [22] and the Eysenck Personality Inventory, extraversion subscale [23]. In addition, pre-study sleep duration, onset and offset (determined by wrist actigraphy for one week prior to study entry) and circadian rhythm phase markers (Morningness-Eveningness chronotype [24] and actigraphic sleep midpoint) were not significantly different across genotypes.

**Table 1:** Characteristics of *PER3^4/4^*, *PER3^4/5^* and *PER3^5/5^* Subjects (Mean±SD).

Characteristic	*PER3* ^4/4^	*PER3* ^4/5^	*PER3* ^5/5^	p*
**N**	52	63	14	
**Age**	31.2±7.0	28.9±6.3	29.7±8.2	0.191
**BMI (kg/m^2^)**	25.50±3.54	24.19±3.57	23.75±2.39	0.076
**Sex (M/F)**	28/24	30/33	5/9	0.473
**Ethnicity (Caucasian/African American/Other)** #	20(.41)/31(.41)/1(.25)	24(.49)/37(.49)/2(.50)	5(.10)/8(.10)/1(.25)	
**Morningness-Eveningness Composite Scale**	40.47±5.63^a^	39.00±5.44	41.86±6.50	0.154
**Beck Depression Inventory (BDI)**	1.35±1.96^b^	1.69±2.51^d^	1.14±2.03	0.600
**Eysenck Personality Inventory (Extraversion subscale)**	15.16±4.04^c^	15.73±3.64^e^	14.50±4.03	0.505
**North American Adult Reading Test (IQ)**	107.10±8.61^c^	105.30±7.97^f^	104.57±6.69	0.410
**Sleep Onset** **	23:49±0.93 h^c^	23:58±0.92 h	23:40±0.85 h	0.462
**Sleep Offset** **	07:46±0.90 h^c^	08:00±1.00 h	07:40±0.85 h	0.296
**Sleep Midpoint** **	03:58±0.35 h^c^	04:01±0.35 h	03:59±0.27 h	0.826
**Total Sleep Time** ** **(Sleep Duration)**	7.96±0.73 h^c^	8.06±0.74 h	8.00±0.55 h	0.780

a n = 49.

b n = 51.

c n = 50.

d n = 62.

e n = 59.

f n = 60.

* p values are for the comparison of the three genotypes.

** Determined by wrist actigraphy (one week prior to study entry).

# Genotypic frequencies are in parentheses. 4-repeat allele frequency was 650 and 5-repeat allele frequency was 350 for both Caucasians and African Americans.

### Cognitive Performance

**Figure 1:**
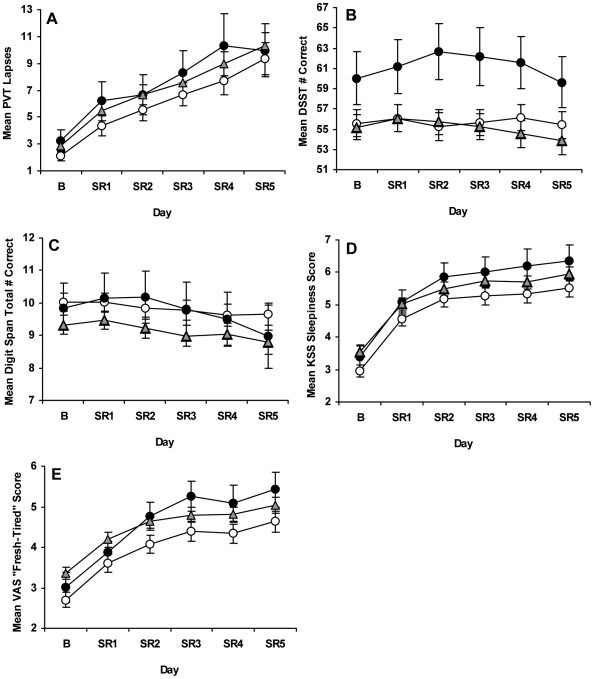
Neurobehavioral performance at baseline and during chronic partial sleep deprivation for the *PER3* groups. Mean (±SEM) (A) PVT lapses (>500 ms reaction times) per trial, (B) total number correct per trial on the Digit Symbol Substitution Task (DSST) and (C) Digit Span (DS) task, and scores per trial on the (D) Karolinska Sleepiness (KSS) and (E) “Fresh-Tired” Visual Analog Scale (VAS) at baseline (B) and each partial sleep deprivation/restriction night (SR1-SR5) for *PER3^4/4^ (open circles)*, *PER3^4/5^ (gray triangles)* and *PER3^5/5^ (closed circles)* subjects. Although all genotypes showed increased PVT lapses and variability across chronic PSD, there were no differential responses in lapses nor did one genotype show more lapses than the other groups at baseline or during chronic PSD. *PER3^5/5^* subjects had better cognitive throughput than their *PER3^4/4^* counterparts, as indicated by significantly higher DSST scores across days; there were no differential changes in DSST scores across chronic PSD or significant changes across days. For all groups, DS total correct scores significantly decreased, and KSS and VAS scores significantly increased across chronic PSD, but there were no differential changes or group differences in these measures during chronic PSD.

Chronic PSD induced significant deficits in neurobehavioral performance across days as shown by increases in lapses (>500 ms reaction times) on the Psychomotor Vigilance Test [PVT; 25]–[27], a well-validated vigilant attention task (Figure 1A), and by increasing variability for all 3 groups across chronic PSD days (Figure 1A). Although all genotypes significantly increased the number of lapses across days (*F*_2.46, 310.29_ = 55.35, p<0.001), there were no differential responses in PVT lapses (day×genotype: *F*_4.93, 310.29_ = 0.31, p = 0.907) nor did one group show more lapses than the other groups across days (genotype: *F*_2,126_ = 0.67, p = 0.514). Moreover, PVT lapses showed no significant baseline (*F*_2,126_ = 0.63, p = 0.533) or sleep deprivation (*F*_2,126_ = 1.23, p = 0.296) differences (Table 2).

**Table 2:** Cognitive and Sleepiness Measures at Baseline and Across Sleep Restriction in *PER3^4/4^, PER3^4/5^* and *PER3^5/5^* Subjects (Mean±SD).

Measure	Baseline							Sleep Restriction (SR1-SR5)						
	*PER3^4/4^*		*PER3^4/5^*			*PER3^5/5^*	p*	*PER3^4/4^*		*PER3^4/5^*			*PER3^5/5^*	p*
***Cognitive Measures***														
**PVT (lapses)**	2.12±2.75		2.83±3.15		3.27±2.91		0.296	6.55±6.21		7.63±6.05		8.19±5.77		0.533
**DSST (# correct)**	55.59±9.52		55.17±9.70		60.03±9.79		0.229	55.69±9.20		55.17±10.12		61.56±9.58		0.082
**DS Forward (# correct)**	5.56±0.97		5.18±1.04		5.36±1.48		0.181	5.44±0.99		5.06±1.08		5.26±1.61		0.194
**DS Backward (# correct)**	4.47±1.21		4.15±1.46		4.47±1.59		0.423	4.34±1.27		4.03±1.39		4.46±1.46		0.359
**DS Total (# correct)**	10.03±1.98		9.33±2.31		9.83±2.95		0.255	9.78±2.11		9.10±2.33		9.72±2.98		0.259
***Sleepiness Measures***														
**KSS (score)**	2.96±1.31	3.55±1.39		3.39±1.42			0.068	5.13±1.62	5.55±1.51		5.83±1.48			0.200
**VAS (score)**	2.70±1.35	3.35±1.21†		3.01±1.39			0.031	4.83±1.29	4.68±1.34		4.17±1.57			0.118
**MWT** ** **(min)**	22.58±10.02	16.81±10.23†		21.59±11.80			0.044	11.30±9.05	10.49±8.71		12.53±9.60			0.777

* p values are for the comparison of the three genotypes

† Different from *PER3^4/4^*, p≤0.05, Bonferroni correction

** Modified version administered once at Baseline and once at SR5 only; *PER3^4/4^* (n = 33), *PER3^4/5^* (n = 46), *PER3^5/5^* (n = 11).

Abbreviations: Psychomotor Vigilance Test (PVT); Digit Symbol Substitution Task (DSST); Digit Span (DS) task; Karolinska Sleepiness Scale (KSS); “Fresh-Tired” Visual Analog Scale (VAS); Maintenance of Wakefulness Test (MWT).

As shown in Figure 1B, the 3 groups showed no differential changes on Digit Symbol Substitution Task [DSST; 28] performance across chronic PSD (day×genotype: *F*_5.79, 365.01_ = 1.55, p = 0.163) nor did scores change significantly across days (day: *F*_2.90, 365.01_ = 1.55, p = 0.163). Although the DSST did not show 3-group differences (genotype: *F*_2,126_ = 2.37, p = 0.098), comparison of the two homozygote groups only—an approach used by Viola et al. [14]—showed that *PER3^5/5^* subjects had better cognitive throughput than their *PER3^4/4^* counterparts in response to chronic PSD (in contrast to data from Viola et al. [14]), as indicated by significantly higher DSST scores across days (genotype: *F*_1,64_ = 3.96, p = 0.050). These two groups showed no differences in baseline DSST scores (*F*_1,64_ = 2.37, p = 0.128), but *PER3^5/5^* subjects had significantly higher scores across the 5 PSD nights (*F*_1,64_ = 4.40, p = 0.040). By contrast, overall performance on the Digit Span (DS) task, a test of working memory storage capacity [28], significantly decreased across days for all groups (day: *F*_2.78, 349.52_ = 4.51, p = 0.005), although there were no differential changes (day×genotype: *F*_5.55, 349.52_ = 0.59, p = 0.722) or group differences (genotype: *F*_2,126_ = 1.46, p = 0.237) in this measure (Figure 1C). Moreover, DS forward, backward and total correct scores showed no baseline (forward: *F*_2,126_ = 1.73, p = 0.181; backward: *F*_2,126_ = 0.87, p = 0.423; total: *F*_2,126_ = 1.38, p = 0.255) or chronic PSD (forward: *F*_2,126_ = 1.66, p = 0.194; backward: *F*_2,126_ = 1.03, p = 0.359; total: *F*_2,126_ = 1.37, p = 0.259) differences (Table 2).

### Subjective and Physiological Sleepiness

Chronic PSD produced significant increases in subjective sleepiness across days as shown by increases in Karolinska Sleepiness Scale [KSS; 29] scores for all genotypes (Figure 1D; day: *F*_2.96, 373.33_ = 103.23, p<0.001). Despite such increased scores across chronic PSD, there were no differential responses in sleepiness ratings (day×genotype; *F*_5.93, 373.33_ = 0.54, p = 0.778) nor did one group have higher ratings than the other groups across days (genotype: *F*_2,126_ = 1.93, p = 0.150). Scores on the “Fresh-Tired” visual analog scale [VAS; 30] showed similar results (Figure 1E; day: *F*_2.42, 304.95_ = 79.38, p<0.001; day×genotype: *F*_4.84, 304.95_ = 1.20, p = 0.310; genotype: *F*_2,126_ = 2.44, p = 0.090). Notably, neither self-rating scale showed chronic PSD differences across genotypes (KSS: *F*_2,126_ = 1.63, p = 0.200; VAS: *F*_2,126_ = 2.18, p = 0.118; Table 2). While the KSS showed no baseline group differences (*F*_2,126_ = 2.75, p = 0.068), the VAS showed a small but reliable difference at baseline (*F*_2,126_ = 3.59, p = 0.031), with *PER3^4/5^* subjects rating themselves as more fatigued than *PER3^4/4^* subjects (Bonferroni correction, p = 0.025).

Substantiating the self-rated sleepiness data, a modified version of the Maintenance of Wakefulness Test [MWT; 17], [31]—a measure of the ability to resist sleep—also did not differ significantly across groups or show differential changes to chronic PSD, although all three genotypes were significantly less able to resist sleep as a result of chronic PSD (day: *F*_1,87_ = 35.71, p<0.001; day×genotype: *F*_2,87_ = 1.72, p = 0.186; genotype: *F*_2,87_ = 2.11, p = 0.130). The groups did not differ across chronic PSD (*F*_2,87_ = 0.25, p = 0.777; Table 2), but showed a slight but reliable difference at baseline (*F*_2,87_ = 3.24, p = 0.044), with the *PER3^4/5^* group showing a reduced ability to resist sleep compared with the *PER3^4/4^* group (Bonferroni correction, p = 0.050).

### Executive Function Measures

**Figure 2:**
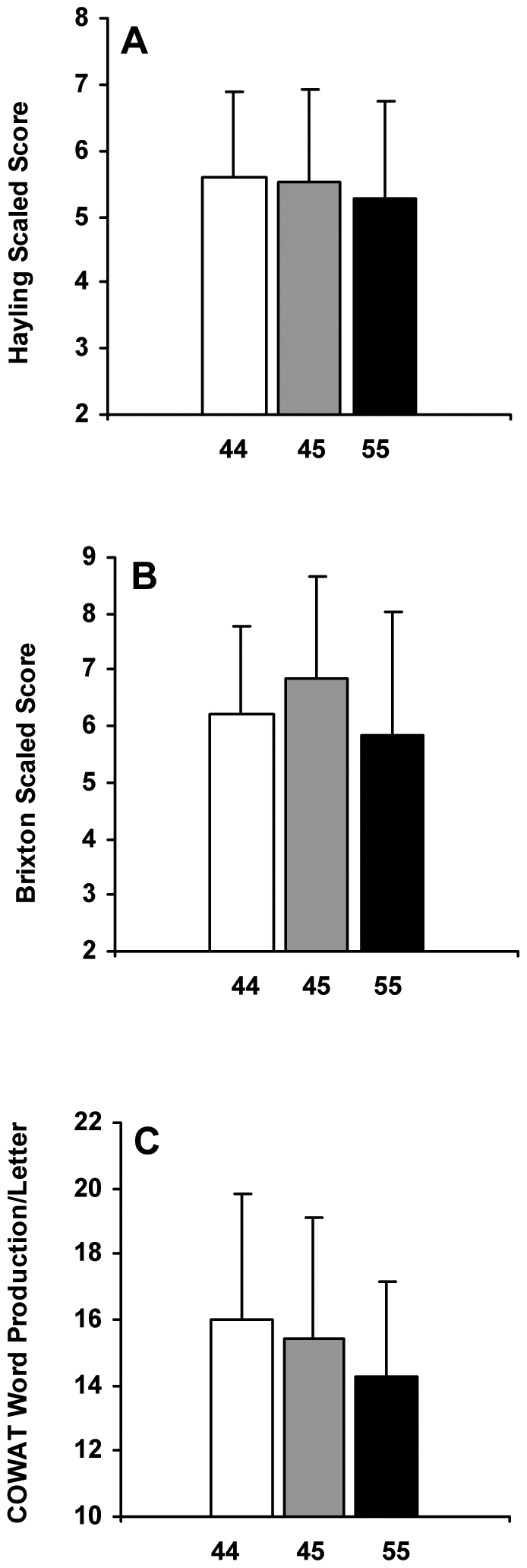
Executive function task performance after five nights of partial sleep deprivation for the *PER3* groups. Mean (±SD) (A) Hayling scaled scores, (B) Brixton scaled scores and (C) Controlled Oral Word Association Test (COWAT) word production per letter at SR5 for *PER3^4/4^* (A: n = 47; B: n = 48; C: n = 46), *PER3^4/5^* (A: n = 50; B: n = 52; C: n = 47) and *PER3^5/5^* (n = 11) subjects. Higher scores indicate better performance on all tasks. None of these executive function tests showed significant differences across the *PER3* genotypes (ps >0.05).

**Figure 3:**
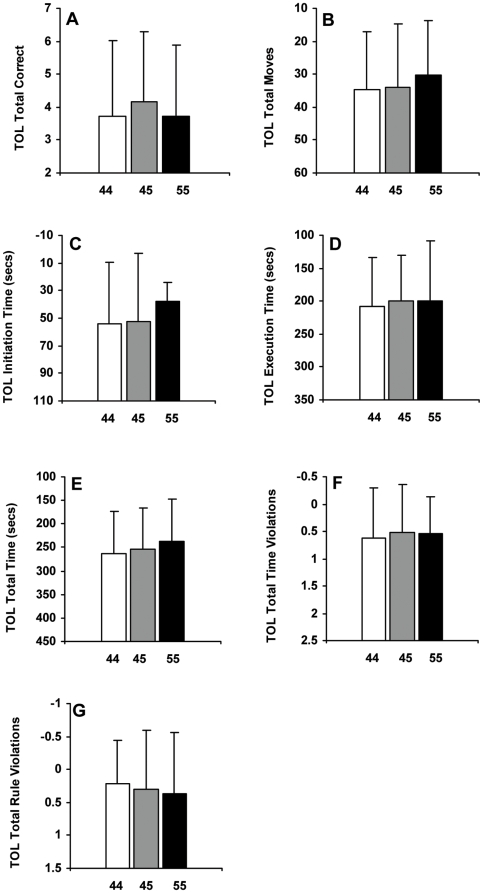
Tower of London performance after five nights of partial sleep deprivation for the *PER3* genotypes. Mean (±SD) Tower of London (TOL) performance measures at SR5 for *PER3^4/4^* (n = 46), *PER3^4/5^* (n = 53) and *PER3^5/5^* (n = 11) subjects. (A) total correct; (B) total moves; (C) initiation time (secs); (D) execution time (secs); (E) total time (secs); (F) total time violations; (G) total rule violations. In all panels except (A), lower values indicate better performance; thus, the top of the ordinate represents better performance. None of the TOL performance measures showed significant differences across the *PER3* genotypes (ps >0.05).

The Hayling [32; Figure 2A; *F*_2,105_ = 0.25, p = 0.779], the Brixton [32; Figure 2B; *F*_2,108_ = 2.28, p = 0.107] and the Controlled Oral Word Association Tests [33; COWAT; Figure 2C; *F*_2,101_ = 0.98, p = 0.379]—all executive function tests measured at SR5—failed to show significant differences across the *PER3* genotypes. Similarly, the 3 *PER3* genotypes did not significantly differ on measures derived from the Tower of London (TOL), an executive function test that assesses planning abilities [34; Figure 3; A: *F*_2,107_ = 0.57, p = 0.566; B: *F*_2,107_ = 0.26, p = 0.770; C: *F*_2,107_ = 0.61, p = 0.543; D: *F*_2,107_ = 0.16, p = 0.855; E: *F*_2,107_ = 0.43, p = 0.654; F: *F*_2,107_ = 0.17, p = 0.845; G: *F*_2,107_ = 0.21, p = 0.809].

### Sleep Physiology

The *PER3* variants did not differ significantly in any polysomnographic (PSG) sleep measure at baseline, at SR1 or at SR5 (Table 3). Across genotypes, however, PSG variables displayed an acute response to chronic PSD in a manner consistent with sleep loss and concomitant increases in homeostatic drive. Specifically, sleep efficiency, amounts of stages 3 and 4 (slow-wave) sleep, and amount of REM sleep significantly increased with chronic PSD; by contrast, TST, sleep onset latency, REM latency, WASO, and amounts of stage 1 and stage 2 sleep significantly decreased with chronic PSD (Table 3). There were no differential changes in PSG measures in response to chronic PSD and no reliable main effects of genotype for any PSG variable.

**Table 3:** Polysomnographic Sleep Measures during Baseline, SR1 and SR5 for *PER3^4/4^, PER3^4/5^* and *PER3^5/5^* Subjects (Mean±SD).

Sleep Measure	Baseline Night				Sleep Restriction Night 1 (SR1)				Sleep Restriction Night 5 (SR5)			
	*PER3^4/4^*	*PER3^4/5^*	*PER3^5/5^*	p*	*PER3^4/4^*	*PER3^4/5^*	*PER3^5/5^*	p*	*PER3^4/4^*	*PER3^4/5^*	*PER3^5/5^*	p*
**Total sleep time (min)**	513.90±54.69	513.42±59.34	521.04±45.63	0.904	224.45±9.67	226.62±8.93	226.93±7.16	0.421	233.11±5.10	231.45±8.87	234.12±4.05	0.319
**Sleep efficiency (%)**	85.71±9.10	86.08±9.77	88.42±7.95	0.646	93.69±4.02	94.48±3.72	94.82±2.62	0.455	97.13±2.13	96.35±3.72	95.58±1.71	0.245
**Latency to sleep onset (min)**	15.81±22.75	21.75±18.82	9.65±8.42	0.088	3.71±5.27	3.43±4.20	3.11±2.99	0.894	1.65±3.19	2.52±3.64	1.81±3.11	0.399
**Latency to REM sleep (min)**	73.76±37.21	77.71±36.07	67.85±32.60	0.645	50.51±29.52	55.31±29.74	58.89±26.71	0.556	40.55±26.13	42.50±28.78	45.62±19.30	0.817
**WASO (min)**	58.24±52.83	56.32±54.11	53.92±44.69	0.961	7.29±8.97	5.90±7.27	5.86±5.64	0.640	2.79±2.70	3.57±5.56	2.77±3.61	0.619
**Stage 1 duration (min)**	54.59±23.47	51.74±28.25	58.38±36.28	0.699	14.63±7.83	15.26±11.28	13.64±8.83	0.846	8.98±5.57	8.72±6.10	9.42±7.56	0.925
**Stage 1 (%TST)**	10.63±4.50	10.17±5.44	11.26±6.86	0.770	6.58±3.65	6.84±5.06	6.06±4.02	0.838	3.86±2.43	3.81±2.74	4.03±3.21	0.964
**Stage 2 duration (min)**	272.02±45.24	277.42±56.41	278.42±50.79	0.846	99.82±29.32	99.19±27.78	103.43±34.88	0.890	102.38±27.65	97.49±33.34	90.46±33.07	0.426
**Stage 2 (%TST)**	53.09±7.58	54.02±9.02	53.55±8.89	0.856	44.50±13.08	43.87±12.42	45.96±15.77	0.868	43.94±11.85	42.26±14.63	38.76±14.55	0.454
**Stage 3 duration (min)**	41.85±16.54	39.02±18.84	46.42±21.15	0.384	28.96±11.24	32.94±15.36	34.11±13.53	0.245	33.39±14.21	31.05±14.77	38.65±17.93	0.242
**Stage 3 (%TST)**	8.21±3.41	7.66±3.73	8.88±3.80	0.489	12.88±5.01	14.54±6.70	14.98±5.90	0.286	14.33±6.13	13.38±6.34	16.51±7.60	0.272
**Stage 4 duration (min)**	25.33±25.02	28.41±27.87	21.27±26.67	0.644	30.00±24.74	28.26±27.50	31.50±32.67	0.903	30.24±25.54	35.19±29.74	40.38±39.29	0.464
**Stage 4 (%TST)**	4.78±4.64	5.62±5.66	3.91±4.75	0.488	13.34±10.91	12.39±11.94	13.73±14.08	0.889	12.94±10.89	15.10±12.70	17.12±16.63	0.474
**SWS duration (min)**	67.18±35.14	67.43±38.08	67.69±44.17	0.999	58.96±27.81	61.20±30.23	65.61±32.70	0.752	63.63±28.30	66.25±30.95	79.04±37.44	0.272
**SWS (%TST)**	12.98±6.52	13.28±7.65	12.79±7.68	0.966	26.22±12.33	26.93±13.05	28.71±14.05	0.815	27.26±12.07	28.47±13.13	33.62±15.72	0.292
**REM duration (min)**	120.09±30.31	116.79±30.64	116.54±20.30	0.836	51.00±15.68	50.90±15.91	44.32±15.73	0.341	58.03±17.13	57.82±15.25	55.96±17.35	0.918
**REM sleep (%TST)**	23.30±5.03	22.52±4.64	22.40±3.63	0.669	22.67±6.75	22.40±6.77	19.51±6.79	0.290	24.89±7.32	24.95±6.44	23.58±7.39	0.806

* p values are for the comparison of the three genotypes. At baseline, *PER3^4/4^* (n = 47), *PER3^4/5^* (n = 57), and *PER3^5/5^* (n = 13); at SR1, *PER3^4/4^* (n = 49), *PER3^4/5^* (n = 54), and *PER3^5/5^* (n = 14); at SR5, *PER3^4/4^* (n = 51), *PER3^4/5^* (n = 57) and *PER3^5/5^* (n = 13).

Abbreviations: Total Sleep Time (TST); Wake After Sleep Onset (WASO); Slow-Wave Sleep (SWS); Rapid Eye Movement (REM).

**Figure 4:**
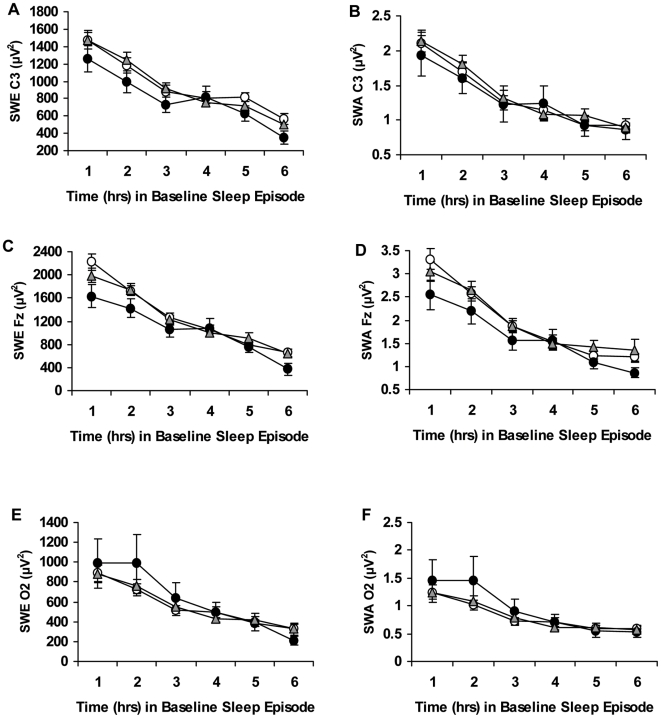
Hourly slow-wave energy and slow-wave activity during baseline for the *PER3* genotypes. Mean (±SEM) hourly slow-wave energy (SWE) and slow-wave activity (SWA) derived from the C3 (A, B), Fz (C, D) or O2 (E, F) channels at baseline for *PER3^4/4^ (open circles)*, *PER3^4/5^ (gray triangles)* and *PER3^5/5^ (closed circles)* subjects. SWE and SWA significantly dissipated across the baseline night for all 3 channels, but not in a differential pattern across genotypes. In addition, the genotypes did not show significant differences in SWE or SWA derived from the C3, Fz or O2 channels.

Similarly, across the baseline night, the three genotypes did not differ significantly in slow-wave energy (SWE) of the delta band in NREM sleep calculated from the C3 (Figure 4A; genotype: *F*_2,89_ = 0.79, p = 0.458), Fz (Figure 4C; genotype: *F*_2,95_ = 1.91, p = 0.154) and O2 (Figure 4F; genotype: *F*_2,98_ = 0.20, p = 0.822) EEG derivations. SWE significantly dissipated across the baseline night for all channels (C3: hour: *F*_3.15, 280.59_ = 101.28, p<0.001; Fz: hour: *F*_2.49, 236.41_ = 85.55, p<0.001; O2: hour: *F*_1.92, 188.45_ = 44.19, p<0.001), but not in a differential pattern across genotypes (C3: hour×genotype: *F*_6.31, 280.59_ = 1.68, p = 0.123; Fz: hour×genotype: *F*_4.98, 236.41_ = 2.18, p = 0.119; O2: hour×genotype: *F*_3.85, 188.45_ = 1.72, p = 0.210).

Slow-wave activity (SWA) also showed no significant differences across the baseline night as calculated from the C3 (Figure 4B; genotype: *F*_2,89_ = 0.09, p = 0.913), Fz (Figure 4D; genotype: *F*_2,95_ = 1.09, p = 0.340) and O2 (Figure 4F; genotype: *F*_2,98_ = 0.34, p = 0.714) EEG derivations. SWA significantly dissipated across the baseline night for all channels (C3: hour: *F*_2.82,__250.78_ = 88.21, p<0.001; Fz: hour: *F*_1.93, 183.27_ = 55.18, p<0.001; O2: hour: *F*_1.81, 177.61_ = 36.91, p<0.001), but not in a differential pattern across genotypes (C3: hour×genotype: *F*_5.64, 250.78_ = 1.17, p = 0.326; Fz: hour×genotype: *F*_3.86, 183.27_ = 1.22, p = 0.304; O2: hour×genotype: *F*_3.63, 177.61_ = 1.40, p = 0.239).

**Figure 5:**
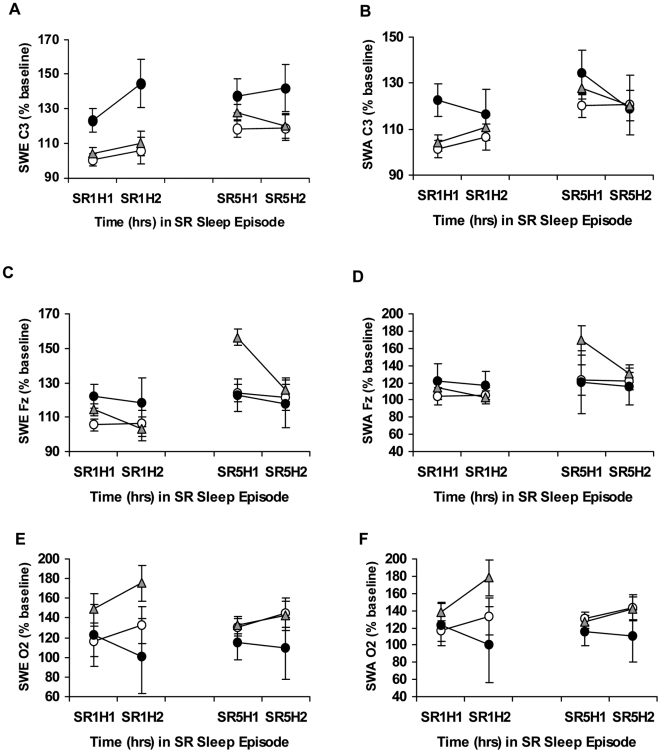
Slow-wave energy and slow-wave activity during chronic partial sleep deprivation for the *PER3* genotypes. Mean (±SEM) hourly slow-wave energy (SWE) and slow-wave activity (SWA) as a percentage of baseline at the same corresponding hour derived from the C3 (A, B), Fz (C, D) or O2 (E, F) channels at partial sleep deprivation/restriction night 1 (SR1) and partial sleep deprivation/restriction night 5 (SR5) for hour 1 (H1) and hour 2 (H2) in *PER3^4/4^ (open circles)*, *PER3^4/5^ (gray triangles)* and *PER3^5/5^ (closed circles)* subjects. SWE derived from C3 (but not from Fz or O2) was significantly higher during chronic PSD in *PER3^5/5^* compared with *PER3^4/4^* and *PER3^4/5^* subjects.

SWE and SWA displayed acute responses to chronic PSD in all genotypes, as evidenced by percentage increases above the corresponding baseline hour for all channels (Figure 5). SWE % baseline and SWA % baseline derived from C3 showed significant changes across chronic PSD (SWE % baseline, hour: *F*_1.87, 181.12_ = 4.41, p = 0.015; SWA % baseline, hour: *F*_2.07, 200.29_ = 4.99, p = 0.007; Figure 5A, 5B), as did SWE % baseline and SWA % baseline derived from Fz (SWE % baseline, hour: *F*_2.29,217.75_ = 4.55, p = 0.009; SWA % baseline, hour: *F*_1.79,172.00_ = 3.07, p = 0.050; Figure 5C, 5D) while measures derived from the O2 channel were not significant (SWE O2 % baseline, hour: *F*_2.27,215.19_ = 0.16, p = 0.874; SWA O2 % baseline, hour: *F*_2.15,201.80_ = 0.30, p = 0.756; Figure 5E, 5F).

SWE % baseline and SWA % baseline failed to show significant differential changes across chronic PSD for the *PER3* genotypes in the C3 EEG derivation (SWE % baseline: hour×genotype: *F*_3.73,181.12_ = 3.73, p = 0.717; SWA % baseline: hour×genotype: *F*_4.13,200.29_ = 0.51, p = 0.732; Figure 5A, 5B), Fz (SWE % baseline: hour×genotype: *F*_4.58,217.75_ = 2.19, p = 0.062; SWA % baseline: hour×genotype: *F*_3.58,172.00_ = 2.00, p = 0.105; Figure 5C, 5D), or O2 EEG derivations (SWE % baseline: hour×genotype: *F*_4,53_ = 1.05, p = 0.388; SWA % baseline: hour×genotype: *F*_4.29,201.80_ = 1.08, p = 0.369; Figure 5E, 5F). SWE % baseline derived from the C3 channel was slightly but significantly higher during chronic PSD for *PER3^5/5^* compared with *PER3^4/4^* and *PER3^4/5^* subjects (SWE % baseline: genotype: *F*_2,97_ = 4.45, p = 0.014; Bonferroni correction, p<0.05; Figure 5A). There were no significant 3-group differences detected in SWA % baseline derived from C3 (SWA % baseline: genotype: *F*_2,97_ = 1.09, p = 0.341; Figure 5B), or in SWE % baseline and SWA % baseline derived from the Fz (SWE % baseline: genotype: *F*_2,95_ = 0.43, p = 0.651; SWA % baseline: genotype: *F*_2,96_ = 0.76, p = 0.471; Figure 5C, 5D) or O2 channels (SWE % baseline: genotype: *F*_2,95_ = 1.81, p = 0.170; SWA % baseline: genotype: *F*_2,94_ = 1.51, p = 0.227; Figure 5E, 5F).

## Discussion

During chronic partial sleep deprivation, *PER3^5/5^* subjects exhibited slightly but reliably higher slow-wave activity and slow-wave energy than *PER3^4/4^* subjects. All three *PER3* VNTR genotypes, however, demonstrated robust and equivalent cumulative decreases in cognitive performance and physiological alertness, and cumulative increases in sleepiness across chronic PSD, with increasing daily inter-subject variability. The genotypes showed no differences at baseline in habitual sleep, physiological sleep structure, circadian phase, physiological sleepiness, cognitive performance, or subjective sleepiness. Thus, collectively, we conclude the *PER3* VNTR polymorphism is not associated with differential vulnerability to the neurobehavioral effects of chronic PSD, although it is related to sleep homeostatic responses. Consequently, this polymorphism may be critical to behavioral performance only when sleep is entirely absent at a specific circadian phase in the early morning hours (i.e., from 6–8 am). Moreover—since the genotypes were comparable at baseline and showed equivalent inter-individual vulnerability to partial sleep deprivation—*PER3* does not contribute to the cumulative neurobehavioral effects of chronic partial sleep loss.

Our chronic PSD protocol produced robust changes in cognitive performance, standard sleepiness and wakefulness measures, and physiological sleep measures characteristic of cumulative sleep loss studies conducted in various laboratories [3], [8], [9], [18], [20], [35]–[40], thus validating our experimental approach. We detected poorer performance on the DS working memory capacity test, the first study to report such a cumulative change, and on the PVT, with variability of lapses increasing for all genotypes across PSD days, and greater self-rated and physiological sleepiness across the five days of PSD—but not differentially so—in *PER3^5/5^* subjects.

*PER3^5/5^* subjects had slightly but reliably better cognitive throughput than their *PER3^4/4^* counterparts, as indicated by significantly higher DSST scores across all 5 PSD nights. Our data contrast with two TSD studies which found that *PER3^5/5^* individuals showed poorer outcomes on a waking performance composite [14] and on specific executive function tasks, but did not differ on the DSST [15]. Since our version of the DSST contained nine symbols and digits instead of eight, and it was longer, as determined from mean data presented in [15], the reasons for this discrepancy remain unclear, but may be methodological or due to differences produced by TSD versus chronic PSD conditions.

SWA and SWE—putative markers of sleep homeostasis—were higher after chronic PSD. In contrast to Viola et al.'s TSD study [14], however, we found no evidence for differential responses to chronic PSD between *PER3^5/5^* and *PER3^4/4^* subjects; this may be due to the nature of such PSD paradigms, in which mitigation of homeostasis occurs via partial daily sleep recuperation [8], [20]. Since overall homeostatic differences were small between *PER3^5/5^* and *PER3^4/4^* subjects during chronic PSD, and there were no differential increases as a result of sleep loss, *PER3* is not the exclusive genetic determinant of the homeostatic response to chronic PSD. In addition, because homeostatic differences occurred during the biological night, other genetic polymorphisms—including clock gene polymorphisms [41], [42]—may influence differential vulnerability to SWA and SWE changes resulting from chronic PSD.

Our study suggests that *PER3^5/5^* subjects may have higher sleep need under chronic PSD conditions, but not at baseline, in contrast to data from Viola et al. [14]. The baseline discrepancies may be explained by a sleep debt factor inherent to a difference in design between studies. In our study, for the first two nights (baseline), all subjects received 10 h time in bed from 2200–0800 h to reduce any pre-existing sleep debt prior to chronic PSD. Baseline was preceded by average pre-study sleep durations of approximately 8 h in both groups; thus, our examination of baseline sleep was completed under fully-rested homeostatic pressure conditions. By contrast, since subjects in the Viola et al. [14] study had on average .5–1 h less sleep prior to entry (7–7.5 h sleep duration), they were not given any saturation nights, and their baseline sleep was curtailed to between 6.78–7.45 h time in bed (for *PER3^5/5^* and *PER3^4/4^*, respectively), they likely still harbored a significant lingering sleep debt. As such, protocol artifact and differential preexisting sleep debt—rather than true endogenous variations—may explain reported baseline differences in sleep propensity and sleep homeostatic measures between the *PER3^5/5^* and *PER3^4/4^* genotypes.

*PER3^5/5^* subjects showed higher homeostatic pressure during sleep chronic PSD, but not poorer cognitive, executive functioning or subjective sleepiness responses to chronic PSD. Such a separation of responses has been noted in other studies in which the homeostatic sleep responses to chronic PSD and TSD have not been reflected in waking neurobehavioral or cognitive responses [8], [43], [44]. These findings suggest that distinct genetic polymorphisms mediate differential vulnerability to cognitive and subjective sleepiness changes resulting from chronic PSD.

Despite prior studies showing that the *PER3* VNTR polymorphism is associated with diurnal preference and delayed sleep phase syndrome [45]–[48], we found no significant genotype differences in circadian phase as measured by chronotype. Similarly, we found no differences in our other measure of circadian phase, the actigraphic sleep midpoint. Our results concur with more recent reports that utilized both physiological and self-rated circadian measures [14], [15], and they may be due to a lack of power to detect such differences in smaller sample size studies, or may highlight false positives in earlier studies [45]–[48].

Notably, we found no differences in executive functioning following five nights of chronic PSD. The COWAT, which measures verbal fluency and orthographic lexical retrieval, and involves the dorsolateral and prefrontal cortex, showed no differences in word production [49], [50]. The genotypes showed no differences in performance on the Hayling and Brixton tests, which measure basic task initiation speed and response suppression performance, and detection of and ability to follow rules, respectively. Poor performance on these tests is associated with frontal lobe dysfunction and dysexecutive symptoms [32], [51], [52]. Moreover, all groups performed equivalently on various performance outcomes of the TOL, which measures executive planning and problem solving abilities, and involves the prefrontal cortex [53]. Thus, across a variety of executive function tasks, *PER3^5/5^* subjects performed at par with *PER3^4/4^* and *PER3^4/5^* subjects, in contrast to results from a previous study indicating executive functioning impairment in this group [15].

In the Groeger et al. study [15], the performance deficits posited to be mediated by the *PER3* VNTR polymorphism occurred selectively only at two time points—2–4 h after the midpoint of the circadian melatonin rhythm—approximately from 6–8 am. We could not collect data after the 0200 h test bout, since our subjects were asleep from 0400–0800 h on each of the PSD nights. Thus, our results cannot be directly compared with the findings of Groeger et al. [15]. However, such selective findings—both in terms of time of day and executive tests affected—indicate that other genetic polymorphisms may influence executive function and cognitive performance deficits resulting from TSD.

In summary, the *PER3^4/4^*, *PER3^4/5^* and *PER3^5/5^* genotypes demonstrated comparable cumulative increases in sleepiness and cumulative decreases in cognitive performance and physiological alertness, across five nights of chronic PSD, with all genotypes showing increasing daily inter-subject variability. During chronic PSD, *PER3^5/5^* subjects exhibited slightly but reliably higher SWA and SWE than *PER3^4/4^* subjects. In contrast to published data in TSD paradigms, *PER3* polymorphism variants did not differ on baseline sleep measures or in their physiological sleepiness, cognitive, executive functioning or subjective responses to chronic PSD. Thus, the *PER3* VNTR polymorphism is not a genetic marker of differential vulnerability to the cumulative neurobehavioral effects of chronic PSD. We propose that other genes—both circadian and non-circadian—regulate neurobehavioral responses to chronic PSD. Our study is the first to characterize the role of any genetic polymorphism in response to chronic PSD, a condition experienced by millions of individuals persistently and daily due to work, travel and social obligations, and one associated with a wide range of serious health consequences. As such, this study provides a foundation for future studies using a candidate gene approach to investigate neurobehavioral and homeostatic responses to chronic partial sleep deprivation.

## Materials and Methods

### Subjects

One hundred and twenty-nine subjects participated in one of two chronic PSD experiments (described below). Following protocol completion, all subjects were genotyped for the *PER3* VNTR polymorphism [*PER3^4/4^*: n = 52; mean±SD; 31.2 yr±7.0 yr; 24 women; *PER3^4/5^*: n = 63; mean±SD; 28.9 yr±6.3 yr; 33 women; *PER3^5/5^*: n = 14; mean±SD; 29.7 yr±8.2 yr; 9 women; see Table 1]. The percentage of each of our three *PER3* VNTR genotypes approximated those reported by Viola et al. [14]. In order to be eligible for study participation, subjects met the following inclusionary criteria: age range from 22–45 yrs; physically and psychologically healthy, as assessed by physical examination and history; no clinically significant abnormalities in blood chemistry; drug-free urine samples; good habitual sleep, between 6.5–8.5 h daily duration with regular bedtimes, and wake up times between 6–9 am (verified by sleep logs and wrist actigraphy for at least one week before study entry); absence of extreme morningness or extreme eveningness, as assessed by questionnaire [24]; absence of sleep or circadian disorders, as assessed by questionnaire [54] and polysomnography; no history of psychiatric illness and no previous adverse neuropsychiatric reaction to sleep deprivation; no history of alcohol or drug abuse; and no current use of medical or drug treatments (excluding oral contraceptives). The protocols were approved by the Institutional Review Board of the University of Pennsylvania. For all subjects, written informed consent was obtained according to the principles expressed in the Declaration of Helsinki prior to entry; all subjects received compensation for participation.

### Experimental Design

Subjects participated in either an 11 or 16 consecutive day protocol in a controlled environment in the Sleep and Chronobiology Laboratory at the Hospital of the University of Pennsylvania. For the current paper, only data from the first seven nights of the protocols—which were procedurally identical between studies—were analyzed to assess the role of the *PER3* VNTR polymorphism on the effects of chronic PSD. For the first two nights of the study (baseline), subjects received 10 h time in bed from 2200–0800 h to reduce any pre-existing sleep debt; for the subsequent five nights, subjects received 4 h time in bed per night (0400–0800 h) for sleep.

Throughout the experiment, laboratory conditions were highly controlled in terms of environmental conditions and scheduled activities. Ambient light was fixed at <50 lux during scheduled wakefulness, and <1 lux (darkness) during scheduled sleep. Ambient temperature was maintained at 22±1°C. Subjects were continuously monitored by trained staff. Between performance bouts, subjects were restricted from engaging in strenuous activities, although they were allowed to read, play games, watch movies, and interact with laboratory staff to help remain awake (no visitors were permitted). Subjects received three standardized meals per day, plus an optional healthy evening snack. Intake of caffeine, turkey, bananas, alcohol or tobacco was prohibited.

### Neurobehavioral Assessments

Subjects underwent computerized neurobehavioral tests every 2 h during scheduled wakefulness. The neurobehavioral test battery included the following objective and subjective evaluations: the Karolinska Sleepiness Scale (KSS), a Likert-type rating scale of subjective sleepiness [29]; a computerized visual analog scale of fatigue (VAS) anchored by “fresh as a daisy” and “tired to death” [30]; the digit symbol substitution task (DSST), a computerized version of the cognitive performance task bearing the same name in the Wechsler Adult Intelligence Scale [28]; the Digit Span (DS) task, a test of working memory storage capacity, given in both the forward and backward versions [28] and summed to produce a total number correct measure for analysis; and the Psychomotor Vigilance Task (PVT), a cognitive test of sustained attention utilizing reaction times as an assay of behavioral alertness [25]–[27]. Subjects remained seated throughout the neurobehavioral test periods and were behaviorally monitored. Subjects were instructed to perform to the best of their ability and to use compensatory effort to maintain performance. Daily values for each performance task were calculated by averaging scores from all the test bouts that day. Baseline values were determined from the second baseline day's performance.

### Other Measurements

Before the study, subjects filled out a number of questionnaires providing information on various demographics, sleep–wake and circadian-related variables, and psychosocial/personality traits which may predict responses to chronic PSD. These questionnaires included the Morningness-Eveningness Composite Scale [24], the Beck Depression Inventory [22], and the Eysenck Personality Inventory [23]. At baseline, subjects completed the North American Adult Reading Test [21] as a measure of IQ. At SR5, subjects were administered four executive function tests: the Hayling test [32], the Brixton tests [32], the Controlled Oral Word Association Test [33] and the Tower of London [34]. In addition, in the 11-day protocol only, a modified version of the Maintenance of Wakefulness Test [17], [31]—a measure of the ability to resist sleep—was administered once at baseline and once at SR5, with a single MWT trial conducted between 1430–1600 h on these two days. Before each trial, the lights were dimmed to <10 lux and subjects were instructed to “keep your eyes open and try not to fall asleep”. Each trial was terminated at the first occurrence of a microsleep (10 seconds of EEG theta activity) or at 30 minutes if sleep onset was not achieved. If subjects fell asleep according to the microsleep criteria before the end of the 30-minute trial, the number of minutes taken to fall asleep was recorded. Subjects who failed to meet sleep onset criteria received a MWT score of 30 minutes. For the MWT, the final available sample sizes for analysis was as follows: *PER3^4/4^* (n = 33), *PER3^4/5^* (n = 46) and *PER3^5/5^* (n = 11).

### Sleep Architecture

#### Polysomnography

The PSG montage included frontal (Fz), central (C3), and occipital (O2) EEG, bilateral EOG, submental EMG, and ECG. Data were recorded from 2200 h (lights off) to 0800 h (lights on) during the second baseline night, and from 0400 h–0800 h on SR1 and on SR5. Sleep records were visually scored in 30-sec epochs according to standard scoring criteria [55] by a trained scorer. Equipment problems resulted in the loss of some records at baseline, SR1 and SR5. Final sample sizes used for analyses are listed in Table 3 (see footnote).

#### Non-REM EEG SWE and SWA

After artifact rejection, spectral analysis of the sleep EEG was performed with Fast Fourier Transform (FFT) in 5 sec bins. Power spectra were averaged across 30 sec epochs. For each night, slow-wave energy (SWE) in the delta band (0.5–4.5 Hz) was totaled over all epochs of non-REM (visually-scored stages 2–4) sleep. Power in the delta band (SWA) was then calculated by dividing SWE by the number of NREM epochs. These procedures were applied to each of the three EEG derivations (C3, Fz, O2). For the baseline night, absolute values were determined for each hour of sleep for SWE and SWA; for SR1 and SR5, SWE and SWA data were normalized by calculating the percent of the corresponding hour of the baseline night. For some records, EEG signal quality was insufficient or contained too much artifact for reliable power spectral analysis. Thus, the final sample sizes used for hourly analyses were as follows: for baseline C3, *PER3^4/4^* (n = 33), *PER3^4/5^* (n = 47), and *PER3^5/5^* (n = 12); for baseline Fz, *PER3^4/4^* (n = 41), *PER3^4/5^* (n = 46), and *PER3^5/5^* (n = 11); for baseline O2, *PER3^4/4^* (n = 41), *PER3^4/5^* (n = 48), and *PER3^5/5^* (n = 12); for SR1 and SR5 C3, *PER3^4/4^* (n = 41), *PER3^4/5^* (n = 47) and *PER3^5/5^* (n = 12); for SR1 and SR5 Fz, *PER3^4/4^* (n = 43), *PER3^4/5^* (n = 46) and *PER3^5/5^* (n = 10); for SR1 and SR5 O2, *PER3^4/4^* (n = 43), *PER3^4/5^* (n = 44) and *PER3^5/5^* (n = 10).

### Genotyping

Blood samples were collected for genetic analysis on the morning following the second night of baseline sleep. Genomic DNA was extracted from whole blood using Qiagen's QIAamp DNA Blood Mini Kit (Catalog #51106). Genotyping was performed with polymerase chain reaction (PCR) using primers as previously described by [48]. The amplification conditions were as follows: 95°C for 15 minutes, then 30 cycles of 95°C for 1 minute, 60°C for 1 minute, and 72°C for 1 minute followed by 72°C for a 10-minute extension. The genotype of each subject could be unambiguously determined.

### Statistical Analyses

Mixed model analyses of variance (ANOVA), with day or hour as the within-subjects (repeated measures) factor and genotype as the between-group factor, were used to analyze the MWT, PSG, SWA/SWE, PVT, KSS, VAS, DSST and DS data. Greenhouse–Geisser corrections were applied to all within-subjects effects. One-way ANOVA were used to analyze demographic and pre-study measures, executive function measures, and PSG, MWT and cognitive and sleep measures at baseline and during chronic PSD (Tables 2 and 3). Post-hoc comparisons using Bonferroni-adjusted probabilities examined significant group differences for all measures. SPSS Statistical Software, version 15.0 (SPSS Inc., Chicago, IL, USA) was used for statistical analyses; p≤0.05 was considered significant.
